# How do nurse consultant job characteristics impact on job satisfaction? An Australian quantitative study

**DOI:** 10.1186/s12912-017-0246-y

**Published:** 2017-09-11

**Authors:** Michelle Giles, Vicki Parker, Rebecca Mitchell, Jane Conway

**Affiliations:** 1 Centre for Nursing and Midwifery Research, Hunter New England Local Health District, Newcastle, NSW 2300 Australia; 2 School of Health, University of New England, Armidale, 2351 NSW Australia; 3 School of Business and Law, University of Newcastle, Callaghan, Australia

**Keywords:** Nurse consultant, Advanced nursing practice, Job satisfaction

## Abstract

**Background:**

There is a direct link between job satisfaction, nurses’ job performance and improved patient outcomes. Understanding what job characteristics influence job satisfaction is vital if health organizations are to optimize individual employee satisfaction and performance. This is particularly necessary in the Nurse Consultant role, which is a multifaceted role that has evolved to meet the dynamic and changing needs of health services. This study aims to examine how job characteristics influence Nurse Consultant job satisfaction and identify differences across metropolitan and rural contexts.

**Methods:**

This paper presents quantitative findings that are part of a larger prospective cross sectional mixed method study. An online survey consisting of a variety of job characteristic factors was administered to all NCs working in a large Local Health District in New South Wales, Australia over an 8-week period in 2010. Descriptive analysis identified NC’s perceptions of job satisfaction and job characteristics in their current role and factor and regression analysis identified relationships between these factors.

**Results:**

Job satisfaction was identified as high (mean 4.3) and is strongly correlated with job autonomy, role clarity, role conflict and job support. A high level of role clarity has a moderating effect on the relationship between job autonomy and job satisfaction.

**Conclusions:**

Study findings inform how we prepare nurses for the NC role and how managers engage with and support NCs in their role taking into account context.

Understanding the factors that influence job satisfaction and role effectiveness gives managers valuable information to assist in positioning and supporting these roles to maximize effectiveness across integrated and contemporary models of health care delivery.

**Electronic supplementary material:**

The online version of this article (10.1186/s12912-017-0246-y) contains supplementary material, which is available to authorized users.

## Introduction

The Nurse Consultant (NC) role in Australia has existed for almost three decades. The role was created to provide an advanced clinical career pathway for nurses [1]. Since then the role has evolved to meet the dynamic and varied needs of health services. NC type roles have been recognized both in Australia and internationally as providing clinical leadership, providing opportunity for advanced practice nurses to act as change agents, working across and within interprofessional health care teams effecting quality health care outcomes [2–5].

Healthcare organizations are highly regulated and driven by policy and guidelines in order to meet patient safety standards. Within this context, the NC role is described as autonomous, flexible, highly connected and multidimensional [2, 6–9]. This juxtaposition has resulted in lack of clarity about NC roles and their function [10–12]. There has been a reactive rather than strategic approach to development and placement of NC roles and in addition, review of the role and its impact has not kept pace with changing contexts of practice [8]. This has seen the NC’s role being applied inconsistently across health care contexts, contributing to ambiguity regarding the role and its impact and contribution [5, 13, 14]. Lack of role clarity has long been associated with decreased role effectiveness and can lead to role tensions and role conflict and decreased job satisfaction [15]. Hence, understanding what influences job satisfaction and other work behaviours is vital if organizations are to optimize individual employee satisfaction and performance, particularly in this multidimensional advanced practice nursing role. This paper examines the relationship between job satisfaction and other characteristics such as role clarity, role conflict, job autonomy and job support.

## Background

Work attitudes, feelings, beliefs and thoughts that employees have about their role and organization are important antecedents of how employees participate and behave in their work environment [16]. Job satisfaction is an important antecedent, identified as having a direct link with nurse’s job performance [16, 17] and retention [16]. Job satisfaction in nurses has also been directly linked to improved patient satisfaction and outcomes [18]. Job satisfaction is recognised as essential in MAGNET hospital principles where access to support from nurse leaders, job autonomy, opportunity, information and resources are key imperatives [19]. Therefore, understanding what influences job satisfaction and other work behaviours is vital if organisations are to optimize individual employee performance.

Within the literature several theoretical frameworks exist that are relevant to understanding job satisfaction. Role theory and job characteristics theory, both used effectively to better understand job satisfaction and role effectiveness, have been employed in this study. Role theory provides perspectives to explore roles (social positions) through role expectations and enactment [20] and job characteristics theory incorporates concepts such as job autonomy to determine individual work motivation, performance and satisfaction [21, 22]. These elements are integrated and not mutually exclusive, in that role expectations influences role enactment which will be influenced by job characteristics.

### Role theory and job satisfaction

A role is centered around behaviors that are characteristic of persons in context and involves overt actions that may be observed and that characterize the persons observed [23]. Roles can be viewed either as patterns of behavior or as expectations that are presumed to cause those overt actions and patterns. [23].

Roles may also be conceived as limited to a context, to interactions with other persons or to be those behaviors that accomplish functions [23]. The concept of role is interdisciplinary in nature with a dynamic perspective that allows a degree of variability amongst those enacting the same role [20]. Therefore, role theory is appropriate to examine NC roles in the context of interprofessional healthcare teams and collaborative practice [24] and in light of their dynamic and flexible nature [2, 6, 7]. Roles incorporate three levels of society; social position, normative role expectations and role enactment [20]. Social position comes with expectations (normative role expectations) and within an organisational context, the NCs, as employees (social position), must enact their work roles (role enactment) within their organisation so that the organisation can function as a social entity [20]. However, expectations may vary between individuals based on organisational and informal group demands and these demands change over time (Biddle, 1986). Hence, role theory is recommended as useful in examining role characteristics where there is confusion about role functions and tensions creating role conflict [20, 25].

The NC role has traditionally been described with several functions or domains of practice [26] with key role responsibilities varying considerably depending on context [2, 27]. However, the role has become increasing complex over time, being described in current literature as highly collaborative, flexible and multidimensional in nature [2, 5–7, 27] and as being required to span professional and organizational boundaries [2, 5]. The complexity, interdependence and wide-ranging remit of the NC role has set the scene for increasing role ambiguity and confusion about role scope, function and positioning. Clearly for the NC role to be effective there needs to be congruence between role expectations and role enactment. Role clarity is a role theory concept that is reflective of this congruence, particularly relevant to understanding job satisfaction and identified as enhancing individual and team effectiveness [15, 28, 29]. Role clarity, is defined as the extent to which an employee is aware of what responsibilities they carry in their role and what is expected of them [30].

Lack of role clarity, a recurring theme in the literature examining the NC role [10–12] has long been identified to have a negative impact on role conflict and job satisfaction and ultimately on role effectiveness [15, 31]. Having clarity in terms of role purpose and impact provides valuable organizational knowledge in establishing a clear understanding of role responsibilities and expectations to enable better role performance within health care teams [15]. Hence this paper explores the relationship between role clarity, role conflict and job satisfaction with the following hypotheses;

Hypothesis 1: Role clarity is positively related to NC job satisfaction.

Hypothesis 2: Role conflict is negatively related to NC job satisfaction.

### Job characteristics theory and job satisfaction

Job characteristics theory is based on the assumption that certain characteristics determine whether or not jobs provide opportunity for motivation and how positively a person will respond to a complex and challenging job [21, 22]. Hackman et al. [21] posited five core job characteristics (or dimensions): job variety (capacity to use a range of skills in doing a job), job identity (capacity to complete a whole piece of work rather than a sub-component), job significance (significance of the job for others), autonomy (the freedom and discretion afforded to an employee to make decisions regarding work), and feedback (reflecting whether the employee receives direct and clear information about effectiveness of performance) [21, 22]. Two of these characteristics are explored in this paper; job autonomy and feedback.

Job autonomy has consistently been identified as contributing to job satisfaction in nurses [32]. The need for autonomy is likely to be stronger in these advanced practice nurse roles because of their flexible and complex nature [2, 6, 27]. Job autonomy allows NCs to make decisions on the basis of their specialist expertise and experience. Due to the complexity of their work and the variation in job requirements, job autonomy provides the opportunity to fully utilise accrued expertise, effectively solve problems and increase felt responsibility for work outcomes. This leads to the following hypothesis:

Hypothesis 3: Job autonomy is positive related to NC job satisfaction.

Further, the capacity of NCs to effectively utilize their job autonomy is likely to be dependent on the extent to which there is a clear understanding of the purpose and function of their role, reflected in role clarity. Without role clarity, NCs will be less likely to work effectively in achieving personal, team and organizational goals.

As professional groups navigate the process of change there is often a need to explore refine and often redefine roles [33, 34]**.** This is particularly relevant when individuals are challenged to assume new work patterns or environments, as is the case for the NC over the past decade. In order to adequately position and support the role and provide appropriate education and support for these complex roles it is imperative that managers are aware how change has influenced attitudes and job characteristics, and ultimately the effectiveness and fit of the NC role in current health care delivery models. The following is the final hypothesis explored in this paper:

Hypothesis 4: Job autonomy will moderate the positive relationship between role clarity and job satisfaction. This relationship will be such that at higher levels of job autonomy, the positive relationship between role clarity and job satisfaction will be stronger.

Managers are key players in positioning and supporting the NC role for optimal outcomes. Job characteristics and behaviors can be influenced by managerial expectations and interventions [35], so awareness of job characteristics that influence job satisfaction and role effectiveness assists managers in making decisions related to realignment of current roles and positioning and support of new roles for maximum contribution. Managers are in a key position to influence the climate of NCs' job satisfaction in the workplace through support and feedback, communicating clearly, clarifying role expectations and ensuring NCs' have autonomy in their role. This informs the basis for the next hypothesis;

Hypothesis 5: Job support is positively related to NC job satisfaction.

If NC’s are more satisfied in their roles there are clear advantages to the organization as they are more effective in their role and will have enhanced organizational commitment making them more likely to continue in their position [16, 19, 36].

Job satisfaction is particularly relevant in rural contexts where recruitment and retention challenges are well acknowledged [37]. Rural clinicians have been described as more generalist in nature with the need to adjust their work patterns to be flexible with an expanded scope of practice [38]. Many rural clinicians also work in isolation with minimal support structures and networks [39]. Differentiation of NC job characteristics under these circumstances is an important and unique aspect of this study.

This paper examines how role clarity, role conflict, job autonomy, job support all contribute to and/or moderate NC job satisfaction. This information provides a platform for organizations and managers to better understand the NC role and position it for optimal performance and success in health care delivery models.

## The study

### Aim

The aim of this study is to quantify NC job characteristics and detail how these characteristics contribute to NC job satisfaction. In addition, this paper explores differences across metropolitan and rural contexts. This will provide better understanding of role positioning to maximize effectiveness across contexts. This study is part of a larger study [40] aimed at gaining a clearer understanding of the Australian Nurse Consultant role.

### Design

The larger study used a mixed method sequential cross sectional design [40]. The findings presented in this paper are from the online survey conducted in phase 1 of the study.

### Participants and setting

All NCs employed in a large Local Health District (LHD) in New South Wales (NSW), Australia were targeted for participation in the online survey. The LHD is the only one in New South Wales (NSW), Australia that encompasses metropolitan, rural and remote areas within its borders. This is an advantage as it enables rural and metropolitan contexts to be examined. At the time of the survey there were 194 NCs employed within the LHD, 64% (n 124) were located in a metropolitan area. All NCs identified from the workforce database were invited to participate in an online survey via their employee email accounts. To encourage participation two information sessions were held in the LHD prior to circulation of the survey, and reminder emails were circulated to all NCs during the period that the survey was open.

### Data collection

The online survey consisted of 43 questions with likert scale and multiple choice responses. It is included as a Additional file 1 (Online Survey). The survey used was a modified version of that used by Guest et al. [30] in the UK to evaluate the introduction of the role of the Nurse Consultant and Health Visitor. The survey required minor modifications which were done with the permission of the authors. Questions were clustered into sections according to constructs of interest. Reported in this paper are the findings from the sections related to the study variables; perceived job satisfaction, role clarity, role conflict, job autonomy and job support. NC respondents were asked to rate the extent to which they agreed or disagreed (1-strongly disagree to 5- strongly agree) with a series of statements based on their’s and other’s perceptions of their role and the opportunities available to them within their roles. Constructs were then derived by combining multiple statements with similar patterns of response as defined by Guest et al., 2001 [27]. Definitions are outlined in Table 1.

**Table 1:** Study Variables/Constructs

Study Variables/Definition	Survey items included in each Construct/Variable
Job satisfaction: The extent to which respondents liked and were satisfied with their job.	To what extent do you agree or disagree with each of the following statements? *(scale 1-strongly disagree to 5 – strong agree)* • I am often bored with my job (reversed) • Most days I am enthusiastic about my job • I am not happy with my job (reversed) • Overall, I am satisfied with my job.
Role clarity: The extent to which the respondent knows what is expected of them and what their responsibilities are in the job.	How true is each of the following statements about your job? *(scale from 1 – Not at all to 5 - To a great extent)* • I know what my responsibilities are • I know exactly what is expected of me • I have a clear idea of what has to be done in my job
Role conflict: Perceived extent of inconsistent expectations and obligations – conflicting and incompatible demands	How true is each of the following statements about your job? *(scale from 1 – Not at all to 5 - To a great extent)* • People at work make conflicting demands on me • I receive incompatible requests from different people at work • I do things at work which are accepted by one person but not by another
Job autonomy: Perceived degree of autonomy & discretion in decision making about their jobs duties	In your CNC / CMC role, to what extent can you? *(scale from 1 – Not at all to 5 - To a great extent)* • Determine methods you use • Choose what work you do • Vary how you do your work • Plan your own work • Do work in a way you think best
Job support: Perceived level of role support from line manager in the form of constructive feedback, care and concern, assistance with difficult tasks and praise and appreciation.	How much of the following do you normally receive from your line manager? *(Scale from 1 - none to 5 - a great deal)* • Constructive feedback on job performance • Care and concern • Useful information • Help with difficult tasks • Praise and appreciation

Definitions taken from Guest et al., 2001 [27]

### Ethical considerations

Humans Research Ethics approval was granted by the LHD Human Research Ethics Committee (approval no:12/07/18/5.07). Participants were provided with details of the purpose of the study prior to their participation. They were also assured that their privacy would be protected at all times and that participation in the online survey would be anonymous.

### Data analysis

Descriptive analysis was used for demographic data analysis and calculating means for individual measures using IBM SPSS software version 24. Rural and metropolitan group means were compared using t test to assess for differences using a significance level of 0.05.

Our measures use reflectively measured constructs and definitions, items and scales are detailed in Table 1. Cronbach alpha testing was conducted on all measures for internal consistency. Internal consistency describes the extent to which all the items in a test measure the same concept or construct and hence it is connected to the inter-relatedness of the items within the test with a value of 1 indicating that all items are measuring exactly the same latent variable [41]. Correlations were performed on all the study variables and these are displayed in Table 2.

**Table 2:** Study variable’s Cronbach Alpha scores, means, SD and correlations

		Alpha	*M*	*SD*	1	2	3	4	5
1	Job satisfaction	0.75	4.3	0.63	**–**				
2	Role autonomy	0.87	4.1	0.69	.21*	**–**			
3	Role clarity	0.84	4.4	0.67	0.16	.42**	–		
4	Role conflict	0.83	3.6	0.99	.18*	.28**	.18*	–	
5	Job support	0.93	3.2	1.12	.31**	.18*	0.15	.17*	–

*p < **.**05 **p < **.**01

To investigate our hypotheses, we used hierarchical regression analysis and entered our control variables, job satisfaction and the study measures as sequential steps into the regression equation. Moderation was tested using Hayes PROCESS macro and the procedure is described in the Moderator results section of this paper.

### Sample size

There was a response rate of 72% (*N* = 140), more than adequate based on a power level of .80, an alpha of .05, using Green’s [42] determination of regression sample size of *N* > 50 plus 8 for each of the independent variable and controls (*N* = 7). This equates to a required sample size of 106.

### Validity, reliability and rigor

The use of a previously developed and validated survey [30] which incorporates a range of the construct identified in the literature related to exploring the nature and function of the role strengthen data reliability. Cronbach alpha scores for each of the factors measured internal consistency and scale reliability [41] and all factors reported in this paper were between 0.7 and 0.9.

## Results

The mean age of respondents was 48 years (range 30 to 64) and 88% were female. Mean years respondents were employed in the LHD was 18 (range 1–40) and mean years employed in their current role was 8 (range 1 month – 28 years). There were 68% (*N* = 95) of respondents located in metropolitan areas, however over half of them identified as having district wide responsibilities, so there responsibilities were across both metropolitan and rural contexts (*N* = 50). There were 32% (*N* = 45) of respondents located in rural areas with rural responsibilities and the remaining (N = 45) had metropolitan only responsibilities.

Measures related to job characteristics and their definitions taken from Guest et al. [30] are outlined in Table 1. Table 2 provides information on cronback alpha scores, mean, SD and correlation for each of the measuement variables.

Further exploration of these measures included the mean scores being categorized into levels as follows:

- Low level: mean score of 2.5 or less
- Moderate level: Mean score of between 2.6 and 3.9
- High level: Mean score of 4 to 5.

**Fig. 1:**
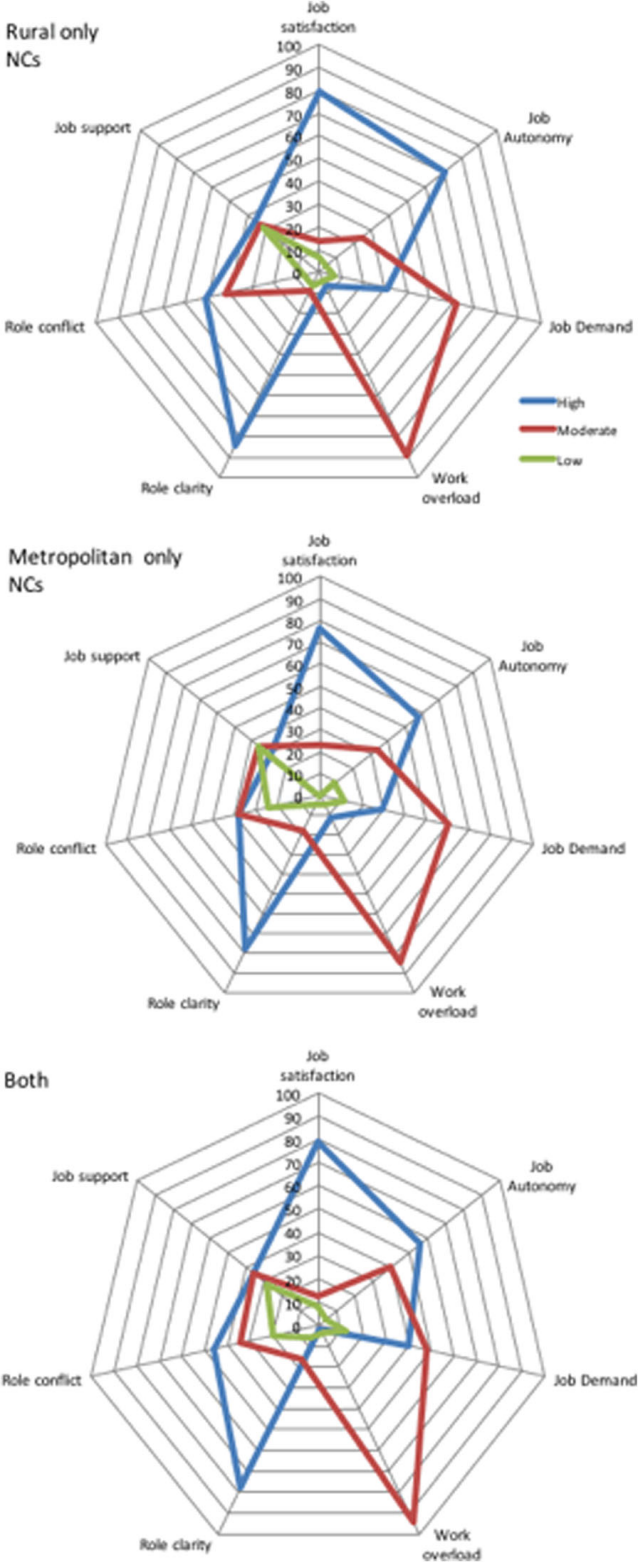
Mean of study variables as levels of Nurse Consultants with rural, metropolitan and both rural and metropolitan responsibilities

The results of this analysis are demonstrated in Fig. 1 and demonstrate similar patterns in the rural, metropolitan and both rural and metropolitan NCs.

### Job satisfaction

Perceived job satisfaction was measured with five individual items. Overall job satisfaction can be reported as positive (mean 4.3) with 76% of respondents rating their job satisfaction as high (Table 2, Fig. 1) with no differences identified across rural and metropolitan context.

### Job autonomy

Job autonomy was also perceived by respondents as high, with an overall mean score of 4.1, 62% of all respondents rating job autonomy as high (Fig. 1). Six percent of respondents perceived they had low job autonomy. Job autonomy was higher in rural areas (mean 4.3) than metropolitan areas (mean 3.9) with 75% of rural NCs rating their job autonomy as high, compared to only 50% of metropolitan based NCs. These differences were not statistically significant.

### Role clarity

Scores reported a generally high level of role clarity (mean 4.3) with 80% of respondents rating role clarity as high (Fig. 1). A higher percentage of rural respondents (90%, mean 4.4) rated role clarity as high compared to metropolitan respondents (75%, mean 4.2), but differences were not statistically significant.

### Role conflict

**Fig. 2:**
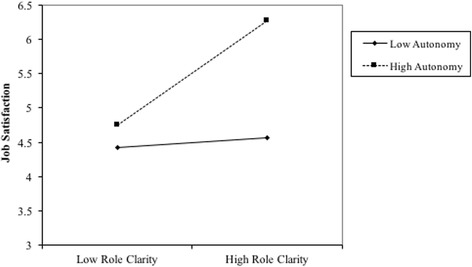
Job autonomy as a moderator between role clarity and job satisfaction

Role conflict with a mean of 3.5 was rated moderate to high. Figure 2 demonstrates that between 30% and 50% of respondents perceived high levels of role conflict, and those in rural only areas expressed significantly higher levels of role conflict (mean 3.9, *p* = 0.015) than those working in metropolitan only areas (mean 3.4).

### Job support

Perceptions of job support were mostly in the moderate range (mean 3.2), 72% of all respondents reporting moderate and high levels of job support, however 28% of respondents did perceived their level of job support as low. There were no differences in perceived support levels between rural and metropolitan respondents, 36% of both groups reporting low levels of support.

Figure 1 highlights the percentage of NCs with low, moderate and high levels of the above study variables related to job characteristics and demonstrates differences between NCs working in metropolitan and rural jurisdictions and those working across both.

### Regression analysis and findings

To investigate our proposed hypotheses, we used hierarchical regression analysis. Using job satisfaction as the dependent variable, we first entered our control variables and subsequently entered our predictor variables, role clarity, job autonomy, role conflict and job support.

The regression equations included the following control variables: consultant age, years in the LHD and years in current role. These variables were included, firstly because age is known as a demographic predictor of job satisfaction [43] The other two control variables years in the LHD and years in their current role is indirectly related to the age variable in that job satisfaction is known to be influenced by job experience, career stage and length of employment tenure [44, 45].

Hypothesis 1 predicts that role clarity will have a significant positive impact on job satisfaction. The findings provide support for this hypothesis (β = .20, *t* = 2.19, *p* = .03, CI = .03 to .56, R^2=^.09).

Hypothesis 2 predicts that role conflict will have a significantly negative impact on NC job satisfaction. This hypothesis is supported by the findings (β = −.23, *t* = −2.48, *p* = .01, CI = −.41 to −.05, R^2^ = .10).

Hypothesis 3 predicts that job autonomy will have a significant positive impact on NC job satisfaction. Again, the findings support this hypothesis (β = .22, *t* = 2.51, *p* = .013, CI = .04 to .35, R^2^ = .09).

Hypothesis 5 predicts that job support will have a significant positive impact on NC job satisfaction. The is again well supported in the findings (β = .27, *t* = 3.02, *p* < .01, CI = .05 to .24, R^2^ = .12).

### Moderators

Moderation was tested using Hayes PROCESS macro and a technique suggested by Edwards and Lambert [46], who suggest testing the relationship at high (1SD above the mean) and low (1 SD below the mean). At 1 SD above the mean for role clarity, the effect of job autonomy on job satisfaction was positive and significant with an effect size of 0.25 (*t* = 2.05, *p* = 0.04, 95% CI = 0.01 to 0.48).

At 1 SD below the mean for role clarity the effect of autonomy on satisfaction was not significant with an effect size of 0.05 (*t* = 0.04, *p* = 0.69, 95% CI = −0.2 to 0.30). Significance of the relationship is supported if the confidence interval does not include zero [47].

Figure 2 represents the moderating effect of a high and low level of job autonomy on the relationship between role clarity and job satisfaction by demonstrating the change in linear relationship (slope) at low, and high levels.

Hypothesis 4, that job autonomy moderates the positive relationship between role clarity and job satisfaction, is well supported in these findings, in that the positive relationship between role clarity and job satisfaction is made stronger.

**Fig. 3:**
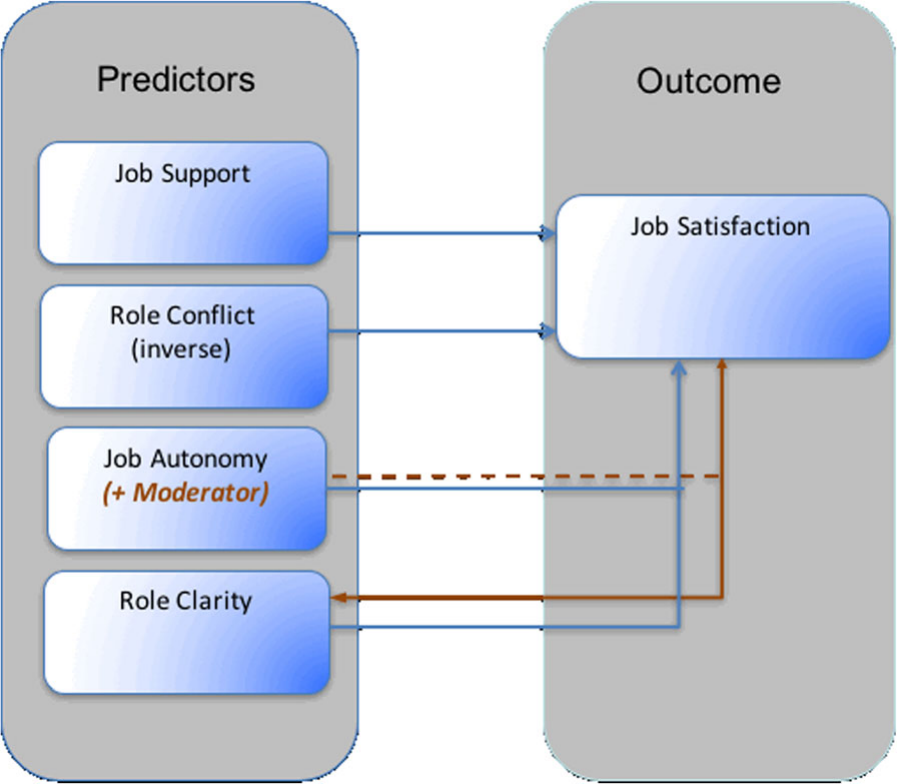
Job characteristics as predictors and antecedents to Nurse Consultant job satisfaction

In summary, all five hypotheses explored in this paper were supported by the findings presented above and are represented diagrammatically in Fig. 3. In Fig. 3, role conflict, role clarity, job autonomy and job support all predict job satisfaction and role autonomy moderates the relationship between role clarity and job satisfaction as depicted by the broken orange line.

## Discussion

This study posited that the relatively unexplored topic, job satisfaction in the advanced practice NC role, is influenced by several key determinants related to characteristics of the NC role. These key determinants are role clarity, role conflict, job autonomy and job support. The findings from this study supported the five hypotheses explored and present valuable and unique insight into the job characteristics that predict job satisfaction in the NC advanced practice role in an Australian health service delivery context, supporting the use of role theory and job characteristic theory as frameworks.

Although job characteristics and job satisfaction have been explored in relation to the new graduate nurse experience [48], there has been limited exploration of these elements in the nursing workforce more broadly, despite recommendations that examining job characteristics is useful where there is confusion about role functions and tensions creating role conflict [20, 25].

In a health care environment under immense pressure to be cost efficient and effective [49, 50], this intelligence is critical in positioning and supporting NCs in a way that maximizes their impact in health care teams. Understanding the determinants of employees’ job satisfaction and hence work performance [16, 17], allows managers to optimize effective functioning of individual’s in their roles [51].

Job satisfaction along with other work characteristics, attitudes and behaviours have been utilized outside the nursing profession as a framework to explore individual attitudes and perceptions across a variety of organizational contexts, so that roles can be positioned and suppotred to optimise performace and outcomes [24]. This approach has been identified as particularly useful where there is lack of role clarity [52] which has been consistently identified as a characteristic of NC type roles [5, 13, 14, 53]. However, it has not been used in an Australian context in examining APN roles, making the findings from this study very noteworthy and timely.

Previous studies have identified job satisfaction as a central component to organizational effectiveness, in particular to employee retention and performance [16]. Further, job satisfaction in nurses has been identified as having a direct link with achieving better patient satisfaction and outcomes and cost effective healthcare [18]. MAGNET health care principles are based on the viewpoint that there is a link between work empowerment and job satisfaction [19]. Two key elements of this empowerment are support from managers and job autonomy, which have both been explored in this paper. The findings from this study strongly support these principles in that job satisfaction increases when there is high role clarity, job autonomy and job support. However further research is needed in this area in nursing.

International literature argues that lack of role clarity in NC type roles has limited its development and standing in current healthcare delivery models [5, 13, 14]. However, the findings from this study indicated that NCs themselves have a clear understanding of their role function and responsibilities, with the majority of NC respondents rating their own role clarity as high. This is of paramount importance as role clarity is identified in our study findings as a strong positive predictor of job satisfaction, having an inverse relationship with role conflict, and working as a moderator between job satisfaction and job autonomy.

Health care organizations are driven by policy and guidelines to meet standards that maintain quality and safety in patient care delivery. Yet service delivery needs to be flexible in order to meet changing needs in complex healthcare environments that incorporate the full spectrum of services from preventive care, across the acute sector and through to end of life care in the home. An individual NC role can span all these sectors [27], so requires flexibility and job autonomy within their role to facilitate this expansive remit. This study's findings report relatively high levels of perceived job autonomy within the role.

Despite job support being identified as a key ingredient in role effectiveness [52], our study findings, similar to findings in Guest et al's [54] evaluation study of the Nurse Visitor role [54], indicate that in many cases the role is not well supported to function in this way. This study’s findings report, in many cases less than optimal job support from managers. Our findings support other studies in this area, where lack of job support is identified as leading to role conflict, decreased job satisfaction and as a consequence less than optimal role performance [31]. Role consensus, or agreement regarding the expectations of role enactment, is also identified as an essential ingredient to optimal role function [15]. However, this need for consensus can fail to account for roles changing overtime and the complexity of function [55]. Findings point to NCs having clarity around their role responsibilities, however more research related to other’s expectations of the role is called for in light of the continued lack of role clarity assigned to the NC role generally [5, 13, 14].

Although there has been ever increasing complexity of health care and enormous changes in health care delivery models there is little research using job satisfaction and other job characteristics related to how change has impacted on nursing roles, particularly related to relationships within integrated inter-professional health care teams. This is despite recommendations that examining role characteristics is useful where there is confusion about role functions and tensions creating role conflict [52]. This is an area of the NC role that requires further research in the future.

Despite the difficulties often encountered by rural clinicians [37, 38], this study found that there were no differences in job satisfaction levels between rural and metropolitan NC’s. However, there were some differences in job characteristics highlighted. Overall there was generally a higher level of perceived role clarity in rural NCs, despite rural practice being identified as more generalist in nature with a broader scope of practice required to meet the holistic needs of isolated populations [38]. Surprisingly, rural NCs had significantly higher levels of role conflict than NCs in metropolitan areas only. This is despite their perceived higher levels of role clarity which contradicts previous findings that identify a link between role clarity and reduced role conflict [31]. Job autonomy, as expected, was higher in rural NCs, and this may be because of their isolation, where at times they function as sole practitioners [38].

The NC role has also been differentiated from other nursing roles due to its leadership, flexibility and autonomy in practice [2, 8, 13]. This has enabled the role in many cases to develop and change with the evolving needs of health care into a highly collaborative role that spans across traditional organizational boundaries. The role function and responsibilities can varys greatly across particular context [27]. For example, many NC roles have care coordinator responsibilities, are embedded within a specialized service and have direct patient care responsibilities. They work within a supportive team environment and their role function and focus is very clear. However, in contrast, many NC roles, particularly those in rural locations are more generalist in nature, cover large geographic distances and the NC can be very isolated in their practice [38], without access to a support network. Others have district wide responsibility for supporting the practice of other clinicians within their specialty area, with very little if any direct patient care [2, 27]. As a result, emphasis on particular aspects of the role varies greatly, and job characteristics such as job autonomy are vital for NC’s to remain responsive to job demands. In line with other studies, this finding is well supported in our study, in that job autonomy and role clarity are strong predictors of job satisfaction [15]. These two job characteristics are rated higher by respondents in rural locations where there is a greater need for flexibility in role function [27, 38].

Health care organizations, systems, processes and ways of working have become increasingly complex over time with factors such as an ageing population and the increased burden of chronic disease seeing models of care evolve and change [56]. There is a strong agenda involving collaborative and inter-professional care and traditional nursing roles have been challenged. It is important to understand the dynamic nature of roles and interaction in developing models of care, and scopes of practice [52, 57]. Current and future NC roles can only be optimized if this understanding is utilized to reduce role ambiguity and role conflict, which will in turn enhance job satisfaction and role effectiveness [5, 15]. NCs are highly skilled senior clinical nurses who contribute a great deal to healthcare delivery [2–5]. Therefore, job satisfaction as a precursor for retention, should be a key consideration for managers and health organisations, and an area for future research particularly in rural locations where retention difficulties are highlighted [37, 38].

## Conclusion

This study’s findings have implications for how managers engage with NCs and their role and also for the nature of relationships and accountabilities taking into account context. To optimize satisfaction in the NC role means grappling with the dynamic between the need for role clarity and the need for job autonomy. This poses a particular challenge within organisations, such as health care, that are highly regulated. More research is needed to understand the functions of the role that require flexibility and how those functions can be supported through clear negotiated expectations. It is likely that one size will not fit all contexts and that other factors such as time in the role will also impact satisfaction. Job satisfaction as an antecedent to retention is particularly relevant in rural contexts. Further research in this area is required.

## Additional file

Additional file 1: Online Survey. (PDF 149 kb)

## References

[1] Chiarella M, Harford E, Lau C, Health N (2007). Report on the evaluation of nurse/midwife practitioner and clinical nurse/ midwife consultant roles.

[2] Giles M, Mitchell R, Parker V (2016). An Australian mixed method study examining nurse consultant connectivity. Nurs Health Sci.

[3] Atsalos C, Biggs K, Boensch S, Gavegan L, Health S, Payk M, Trapolini G (2014). How clinical nurse and midwifery consultants optimise patient care in a tertiary referral hospital. J Clin Nurs.

[4] McIntosh J, Tolson D. Leadership as part of the nurse consultant role: banging the drum for patient care. J Clin Nurs. 2012;18:219–27. 10.1111/j.1365-2702.2008.02520.x 19120750

[5] Humphreys A, Johnson S, Richardson J, Stenhouse E, Watkins M (2007). A systematic review and meta synthesis: evaluating the effectiveness of nurse, midwife/allied health professional consultants. J Clin Nurs.

[6] Cashin A, Stasa H, Gullick J, Conway R, Cunich M, Buckley T. Clarifying clinical nurse consultant work in Australia: a phenomenological study. Collegian. 2014; 10.1016/j.colegn.2014.09.002. 10.1016/j.colegn.2014.09.002 26775527

[7] Lamont S, Brunero S, Lyons S, Foster K, Perry L (2014). Collaboration amongst clinical nursing leadership teams: a mixed-methods sequential explanatory study. J Nurs Manag.

[8] Franks H, Howarth M (2012). Being an effective nurse consultant in the English National Health Service: what does it take? A study of consultants specializing in safeguarding. J Nurs Manag.

[9] Mullen C, Gavin-Daley A, Kilgannon H, Swift J (2011). Nurse COnsultants 10 years on: an insight to the role for nurse managers. J Nurs Manag.

[10] Chang A, Gardner G, Duffield C, Ramus M (2010). A Delphi study to validate an advanced practice nursing tool. J Adv Nurs.

[11] Drennan V, Goodman C (2011). Sustaining innovation in the health care workforce: a case study of community nurse consultant posts in England. BMC Health Serv Res.

[12] Lloyd Jones M (2005). Role development and effective practice in specialist and advanced practice roles in acute hospital settings: systematic review and meta-synthesis. J Adv Nurs.

[13] Jokiniemi K, Pietila A, Kylma J, Haatainen K (2012). Advanced nursing roles: a systematic review. Nurs Health Sci.

[14] Pulcini J, Jelic M, Gul R, Loke A (2010). An international survey on advanced practice nursing education, practice and regulation. J Nurs Scholarsh.

[15] Rheiner N (1982). Role theory: framework for change. Nurs Manag.

[16] Moynihan L, Boswell W, Boudreau J (2000). The influence of job satisfaction and organizational commitment on executive withdrawal and performance.

[17] Gillet N, Colombat P, Michinov E, Pronost A, Fouquereau E (2013). Procedural justice, supervisor autonomy support, work satisfaction, organizational identification and job performance: the mediating role of need satisfaction and perceived organizational support. J Adv Nurs.

[18] Kutney-Lee A, McHugh M, Sloane D, Cimiotti J, Flynn L, Felber Neff D, Aiken L (2009). Nursing: a key to patient satisfaction. Health Aff.

[19] Upenieks V (2003). The interrelationship of organizational characteristics of magnet hospitals, nursing leadership, and nursing job satisfaction. Health Care Manag.

[20] Muuray T. Using role theory concepts to undersyand transitions from hospital -based nursing practice to home care nursing. 1998;29(3):105–11. 10.3928/0022-0124-19980501-05 9652263

[21] Hackman J, Oldham G, Janson R, Purdy K (1975). A new strategy for job enrichment. Calif Manag Rev.

[22] Hackman JR, Oldham GR (1980). Work redesign.

[23] Biddle B (1986). Recent development in role theory. Ann Rev Sociol.

[24] Brookes K, Davidson P, Daly J, Halcomb E (2007). Role theory: a framework to investigate the community nurse role in contemporary health care systems. Contemp Nurse.

[25] Broderick A (1998). Role theory, role management and service performance. J Serv Mark.

[26] Health NSW (2005). Clinical nurse consultant - higher grades - public hospital Nurses’ (state) award. Policy Directive.

[27] Giles M, Parker V, Mitchell R. Understanding Nurse Consultant role engagement in metropolitan and rural contexts. Collegian. 2016. http://dx.doi.org/10.1016/j.colegn.2016.04.002.

[28] Birkinshaw J, Heywood S. Putting organizational complexity in its place. In: Insights & Publications. May 2010 edn. UK: McKinsey & Company; 2010.

[29] Orchard C (2010). Persistent isolationist or collaborator? The nurse’s role in interprofessional collaborative practice. J Nurs Manag.

[30] Guest D, Redfern S, Wilson-Barnett J, Dewe P, Peccei R, Rosenthal P, Evans A, Young C, Montgomery J, Oakley P (2001). A preliminary evaluation of the establishment of nurse, midwife and health visitor consultants: a report to the Department of Health.

[31] Rizzo J, Lirtzman S, House R (1970). Role conflict and ambiguity in complex Organisations. Adm Sci Q.

[32] Bjørk IT, Samdal GB, Hansen BS, Tørstad S, Hamilton GA (2007). Job satisfaction in a Norwegian population of nurses: a questionnaire survey. Int J Nurs Stud.

[33] Major D (2003). Utilising role theory to help employed parents cope with children’s chronic illness. Health Educ Res Theory Pract.

[34] Lambert V, Lambert C (1981). Role theory and the concept of powerlessness. J Psychosoc Nurs Ment Health Serv.

[35] Kahn R, Wolfe D, Quinn R, Snoek J, Rosenthal R (1964). Organizational stress: studies in role conflict and ambiguity.

[36] Kumar BP, Giri VN (2007). Organizational commitment, climate and job satisfaction: an empirical study. ICFAI J Organ Behav.

[37] Wakerman J (2008). Rural and remote public health in Australia: building on our strengths. Aust J Rural Health.

[38] Paliadelis P, Parmenter G, Parker V, Giles M, Higgins I (2012). The challenges confronting clinicians in rural acute care settings: a participatory research project. Rural Remote Health.

[39] Lea J, Paliadelis P, Sanderson H, Thornberry P (2008). The lure of the bush: do rural placements influence student nurses to seek employment in rural settings?. Collegian.

[40] Giles M, Parker V, Mitchell R (2014). Recognising the differences in the nurse consultant role across context: a study protocol. BMC Nurs.

[41] Tavakol M, Dennick R (2011). Making sense of Cronbach alpha. Int J Med Educ.

[42] Green S (1991). How many subjects does it take to do a regression analysis?. Multivar Behav Res.

[43] Bedian A, Ferris G, Kacmar M (1992). Age tenure and job satisfaction: a tale of two perspectives. J Vocational Behav.

[44] Katz R (1980). Time and work: towards an integrative perspective. Res Organ Behav.

[45] White J, Spector P (1987). An investigation of age-related factors in the age-job satisfaction relationship. Psychol Aging.

[46] Edwards J, Lambert L (2007). Methods for integrating moderation and mediation: a general analytical framework using moderated path analysis. Psychol Methods.

[47] Preacher K, Rucker D, Hayes A (2007). Addressing moderated mediation hypotheses: theory, methods, and prescriptions. Multivar Behav Res.

[48] Chang E, Hancock K (2003). Role stress and role ambiguity in new nursing graduates in Australia. Nurs Health Sci.

[49] McCarty M, Fenech B (2013). Towards best practice in national health workforce planning. Med J Aust.

[50] Naccarella L, Freijser L. Boundary Spanners of the Future. In., vol. 23rd September, 2011: The Australian Health Workforce Institute; 2011.

[51] Parker S (2007). That is my job ‘how employees’ role orientation affects their job performance. Hum Relat.

[52] McKenna H, Richey R, Keeney S, Hasson F, Poulton B, Sinclair M (2008). The managerial and development issues of nurses and midwives in new roles. Scandanavian J Caring Sci.

[53] Duffield C, Gardner G, Chang AM, Catling-Paull C (2009). Advanced nursing practice: a global perspective. Collegian.

[54] Guest D, Peccei R, Rosenthal C, Redfern S, Wilson-Barnett J, Dewe P, Coster S, Evans A, Sudbury A (2004). An evaluation of the impact of nurse, midwife and health visitor consultants.

[55] Kerr S (1978). Consensus for change in the role of the learning resources specialist: order and position differences. Sociol Educ.

[56] Welfare AAIoHa: Australia’s Health 2012. In: Australia’s Health series no 13 vol. Cat. no. Aus 156: Australian Government; 2012.

[57] Davidson P, Elliot D (2006). Clinical leadership in contemporary clinical practice: implications for nursing in Australia. J Nurs Manag.

