# Supplementary material for: How do nurse consultant job characteristics impact on job satisfaction? An Australian quantitative study
Source: BMC Nurs. 2017 Sep 11;16:51. doi: 10.1186/s12912-017-0246-y (PMC5594556; doi:10.1186/s12912-017-0246-y)
Supplement: Additional file 1: — Online Survey. (PDF 149 kb) [file 12912_2017_246_MOESM1_ESM.pdf]

# Hunter New England Area Health - Survey for Clinical Nurse / Midwife

## 1. About Yourself

This survey is part of the HNEAHS CNC/CMC Review project. In completing this survey you can be assured of complete anonymity. The survey will take approximately 20-30 minutes to complete. To simplify and strengthen statistical testing, some of the questions ask similar things. It is important that you answer all questions in the questionnaire.

### 1. I am currently employed within HNEAHS as a:

- ◯ CNC
- ◯ CMC

Comments

[ ]

### 2. What is your current age in years?

[ ]

### 3. What is your gender?

- ◯ Female
- ◯ Male

### 4. Please specify your educational qualification from the list below:(may choose more than one answer)

- ☐ Certificate
- ☐ Diploma
- ☐ Bachelor Degree
- ☐ Graduate Certificate
- ☐ Graduate Diploma
- ☐ Masters
- ☐ PhD
- ☐ Professional Doctorate
- ☐ Other

Please specify

[ ]

## 5. Are you currently undertaking any studies:(choose only one answer)

- ◯ Not currently undertaking any studies
- ◯ Certificate
- ◯ Diploma
- ◯ Bachelor Degree
- ◯ Graduate Certificate
- ◯ Graduate Diploma
- ◯ Masters
- ◯ PhD
- ◯ Professional Doctorate
- ◯ Other

Please specify

[ ]

## 6. Do you hold a professional / vocational qualification other than in nursing / midwifery?

- ◯ Yes
- ◯ No

If yes, please specify

[ ]

## 7. How long have you been employed in this Area Health Service?

Years [ ]

Months [ ]

## 8. How long have you been employed as a CNC/ CMC within this Area Health Service?

Years [ ]

Months [ ]

## 2. Your CNC / CMC Role Description

### 1. What is your CNC / CMC position grade?

- One
- Two
- Three
- Ungraded

### 2. Is this your substantive position?

- Yes
- No

### 3. When did you commence in this position?

| | DD | | MM | | YYYY |
|---|---|---|---|---|---|
| CNC / CMC position | | / | | / | |

### 4. Is this CNC / CMC position:

- An ongoing permanent position
- A fixed term project specific position
- The substantive CNC / CMC position no longer exists and I am working in another role

Other (please specify)

### 5. Is your CNC / CMC position:

- A full time position
- A part time position

If part time, how many hours per week.

### 6. If the above position is full time are you job sharing in this role?

- Not applicable
- Yes
- No

If yes, how many are sharing the role (including yourself)

**7. My substantive position is CNC / CMC but I am currently relieving in another position.**

- ◯ Yes
- ◯ No

If Yes, please specify classification of position

**8. Does your CNC / CMC role encompass? (may choose more than one answer)**

- ☐ Rural
- ☐ Metropolitan
- ☐ Both rural and metropolitan
- ☐ Area

**Categories of Health Care need: Source: Modified from Queensland Health Statewide Services Plan 2007 – 2012 & Queensland Health Hubs and Precincts Service Development Framework 2007.**

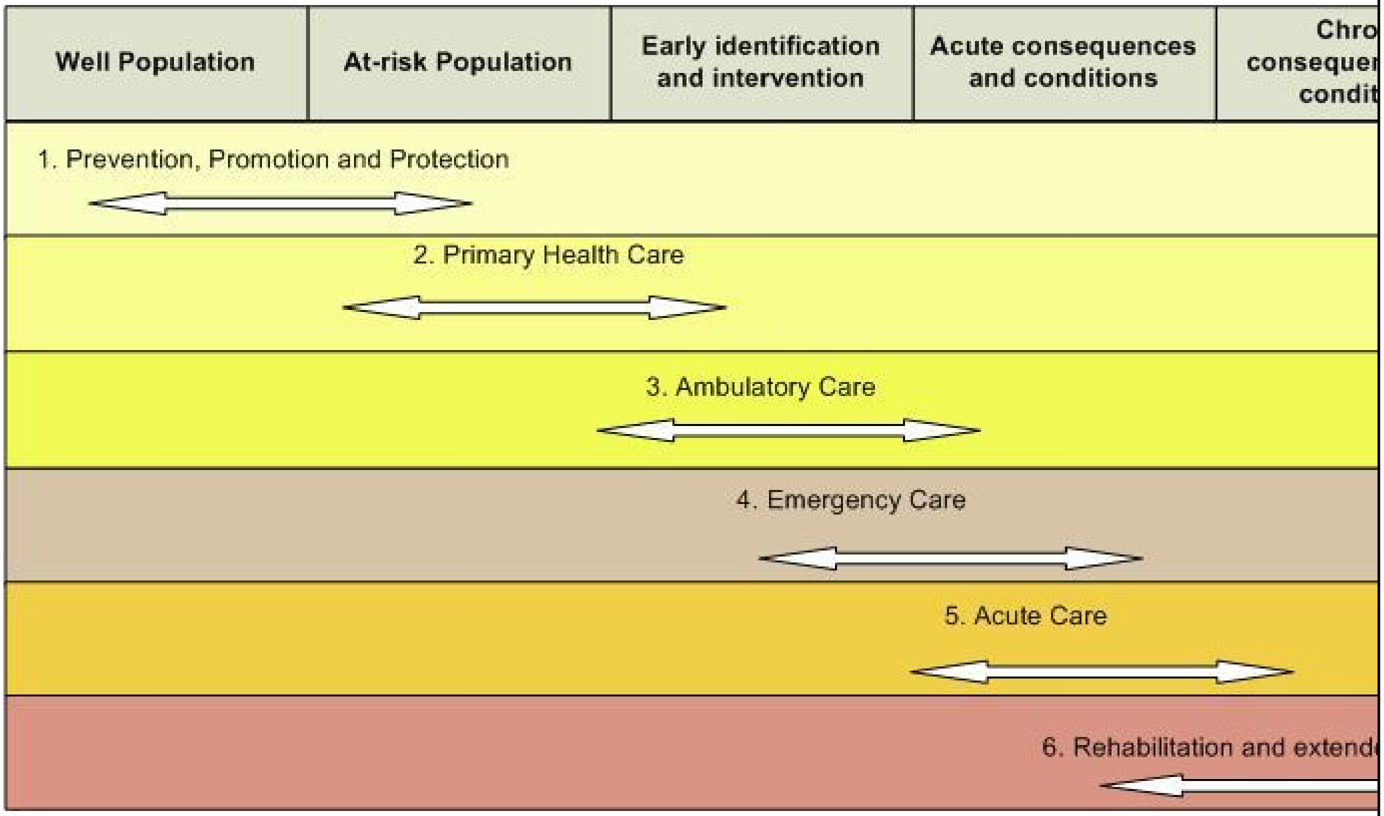

**9. From the above diagram of categories of health care need can you choose the most appropriate percentage of time you spend in each of these categories in your CNC / CMC role ?**

| | 0% | 10% | 20% | 30% | 40% | 50% | 60% | 70% | 80% | 90% | 100% |
|---|---|---|---|---|---|---|---|---|---|---|---|
| Prevention, Promotion and Protection | ○ | ○ | ○ | ○ | ○ | ○ | ○ | ○ | ○ | ○ | ○ |
| Primary Health Care | ○ | ○ | ○ | ○ | ○ | ○ | ○ | ○ | ○ | ○ | ○ |
| Ambulatory Care | ○ | ○ | ○ | ○ | ○ | ○ | ○ | ○ | ○ | ○ | ○ |
| Emergency Care | ○ | ○ | ○ | ○ | ○ | ○ | ○ | ○ | ○ | ○ | ○ |
| Acute care | ○ | ○ | ○ | ○ | ○ | ○ | ○ | ○ | ○ | ○ | ○ |
| Rehabilitation and extended care | ○ | ○ | ○ | ○ | ○ | ○ | ○ | ○ | ○ | ○ | ○ |
| Other | ○ | ○ | ○ | ○ | ○ | ○ | ○ | ○ | ○ | ○ | ○ |

Other (please specify)

**10. How many people report directly to you?**

**11. What is the job title of your immediate Line Manager?**

- ○ Nurse / Midwife Unit Manager
- ○ Nurse / Midwife Manager
- ○ Service Manager
- ○ Service Director
- ○ Director Nursing and Midwifery
- ○ Other

Other (please specify job classification only)

## 12. What is the job title of your immediate Professional Manager?

- ◯ Same as above (Question 11)
- ◯ Nurse / Midwife Unit Manager
- ◯ Nurse / Midwife Manager
- ◯ Service Manager
- ◯ Service Director
- ◯ Director of Nursing and Midwifery
- ◯ Other

Other (please specify)

[ ]

## 13. Role pattern: In a typical week what proportion of your total working time do you usually spend:

| | 0% | 10% | 20% | 30% | 40% | 50% | 60% | 70% | 80% | 90% | 100% |
|---|---|---|---|---|---|---|---|---|---|---|---|
| Working in direct contact with patients / clients? | ◯ | ◯ | ◯ | ◯ | ◯ | ◯ | ◯ | ◯ | ◯ | ◯ | ◯ |
| Collaborating & working in conjunction with colleagues from other professions? | ◯ | ◯ | ◯ | ◯ | ◯ | ◯ | ◯ | ◯ | ◯ | ◯ | ◯ |
| Doing research related activity? | ◯ | ◯ | ◯ | ◯ | ◯ | ◯ | ◯ | ◯ | ◯ | ◯ | ◯ |
| Providing education either formally or opportunistically? | ◯ | ◯ | ◯ | ◯ | ◯ | ◯ | ◯ | ◯ | ◯ | ◯ | ◯ |
| In a management role (ie procuring & allocating resources, staffing and coordinating services) | ◯ | ◯ | ◯ | ◯ | ◯ | ◯ | ◯ | ◯ | ◯ | ◯ | ◯ |

# Hunter New England Area Health - Survey for Clinical Nurse / Midwife

## 14. In your CNC / CMC role how frequently do you interact with each of the following?

| | Daily (1) | 2-3 times weekly (2) | Once per week (3) | Once per fortnight (4) | Once per month (5) | Less than once per month (6) | Never (7) |
|---|---|---|---|---|---|---|---|
| Patients / clients | ◯ | ◯ | ◯ | ◯ | ◯ | ◯ | ◯ |
| Your immediate line manager | ◯ | ◯ | ◯ | ◯ | ◯ | ◯ | ◯ |
| Your immediate professional manager (if different to line manager) | ◯ | ◯ | ◯ | ◯ | ◯ | ◯ | ◯ |
| Colleagues in your own profession | ◯ | ◯ | ◯ | ◯ | ◯ | ◯ | ◯ |
| Junior medical staff | ◯ | ◯ | ◯ | ◯ | ◯ | ◯ | ◯ |
| Senior medical staff | ◯ | ◯ | ◯ | ◯ | ◯ | ◯ | ◯ |
| Allied Health staff | ◯ | ◯ | ◯ | ◯ | ◯ | ◯ | ◯ |
| Senior management staff | ◯ | ◯ | ◯ | ◯ | ◯ | ◯ | ◯ |
| Non clinical professional staff | ◯ | ◯ | ◯ | ◯ | ◯ | ◯ | ◯ |
| Administrative and clerical staff | ◯ | ◯ | ◯ | ◯ | ◯ | ◯ | ◯ |
| Other professional staff | ◯ | ◯ | ◯ | ◯ | ◯ | ◯ | ◯ |
| Clinical Streams / Networks | ◯ | ◯ | ◯ | ◯ | ◯ | ◯ | ◯ |
| People from professional bodies | ◯ | ◯ | ◯ | ◯ | ◯ | ◯ | ◯ |
| University academics - Nursing / Midwifery | ◯ | ◯ | ◯ | ◯ | ◯ | ◯ | ◯ |
| University academics - other disciplines | ◯ | ◯ | ◯ | ◯ | ◯ | ◯ | ◯ |
| Rural staff and services | ◯ | ◯ | ◯ | ◯ | ◯ | ◯ | ◯ |
| People from other external agencies | ◯ | ◯ | ◯ | ◯ | ◯ | ◯ | ◯ |
| State / national committees or working parties. | ◯ | ◯ | ◯ | ◯ | ◯ | ◯ | ◯ |

# Hunter New England Area Health - Survey for Clinical Nurse / Midwife

**15. To what extent are you involved in / make a contribution to each of the following areas of activity?**

| | Not involved at all (1) | Minor involvement (2) | Moderate involvement (3) | Major involvement (4) | I take the lead in this activity (5) |
|---|---|---|---|---|---|
| 1)Making referrals to other professionals | ○ | ○ | ○ | ○ | ○ |
| 2)Prescribing aids or equipment | ○ | ○ | ○ | ○ | ○ |
| 3)Managing complete programs of care | ○ | ○ | ○ | ○ | ○ |
| 4)Developing professional protocols, documentation systems & guidelines | ○ | ○ | ○ | ○ | ○ |
| 5)Monitoring the effectiveness of current therapeutic programs | ○ | ○ | ○ | ○ | ○ |
| 6)Integrating different aspects of practice to improve quality / health outcomes | ○ | ○ | ○ | ○ | ○ |
| 7)Advising and supporting colleagues where standard protocols do not apply | ○ | ○ | ○ | ○ | ○ |
| 8)Advising and supporting colleagues | ○ | ○ | ○ | ○ | ○ |
| 9)Advising and supporting colleagues in rural settings | ○ | ○ | ○ | ○ | ○ |
| 10)Advising and supporting colleagues across this AHS | ○ | ○ | ○ | ○ | ○ |
| 11)Advising and supporting colleagues across the State and / or nationally | ○ | ○ | ○ | ○ | ○ |
| 12)Developing best practice | ○ | ○ | ○ | ○ | ○ |
| 13)Promoting best practice | ○ | ○ | ○ | ○ | ○ |
| 14)Generating and implementing new solutions that will best meet the needs of patients and clients | ○ | ○ | ○ | ○ | ○ |
| 15)Offering expert advice to your own and other professions on care practices, delivery and service development | ○ | ○ | ○ | ○ | ○ |
| 16)Identifying and responding to individual and team education needs | ○ | ○ | ○ | ○ | ○ |
| 17)Consulting with staff across this AHS | ○ | ○ | ○ | ○ | ○ |
| 18)Mentoring staff | ○ | ○ | ○ | ○ | ○ |
| 19)Supervising staff | ○ | ○ | ○ | ○ | ○ |
| 20)Engaging in professional development of staff in my service / unit | ○ | ○ | ○ | ○ | ○ |
| 21)Engaging in professional development of staff across this AHS | ○ | ○ | ○ | ○ | ○ |
| 22)Teaching staff & students in partnership with universities / colleges | ○ | ○ | ○ | ○ | ○ |
| 23)Promoting evidence based practice | ○ | ○ | ○ | ○ | ○ |
| 24) Setting, auditing and monitoring standards | ○ | ○ | ○ | ○ | ○ |
| 25)Evaluating local services against best practice | ○ | ○ | ○ | ○ | ○ |
| 26)Participation as part of a research team | ○ | ○ | ○ | ○ | ○ |
| 27)Undertaking research at a local level | ○ | ○ | ○ | ○ | ○ |
| 28)Leading research teams | ○ | ○ | ○ | ○ | ○ |
| 29)Disseminating knowledge and networking on improving practice | ○ | ○ | ○ | ○ | ○ |
| 30)Developing &/or sustaining new partnerships & networks to improve health outcomes & healthcare delivery systems:- ---a)Locally | ○ | ○ | ○ | ○ | ○ |
| --b)Statewide | ○ | ○ | ○ | ○ | ○ |
| --c)Across primary, secondary & teritary sectors | ○ | ○ | ○ | ○ | ○ |
| | ○ | ○ | ○ | ○ | ○ |

| | | | | | |
|---|---|---|---|---|---|
| --d)Nationally | ◯ | ◯ | ◯ | ◯ | ◯ |
| 31)Developing research protocols | ◯ | ◯ | ◯ | ◯ | ◯ |
| 32)Applying for research funding | ◯ | ◯ | ◯ | ◯ | ◯ |
| 33)Undertaking interventions normally undertaken by medical or other professional staff | ◯ | ◯ | ◯ | ◯ | ◯ |
| 34)Exercising management responsibilities | ◯ | ◯ | ◯ | ◯ | ◯ |
| 35)Representing this AHS & exercising corporate responsibility (ie committee membership, liaising with media). | ◯ | ◯ | ◯ | ◯ | ◯ |

## 16. From the above list of activities (question 15) which are the THREE MOST IMPORTANT activities to you in your CNC / CMC role (please write appropriate activitiy number from question 15)

First most important activitiy

Second most important activity

Third most important activity

## 17. What proportion of your total working time do you usually spend on each of these three activities listed in Question 15?

| | 0% | 10% | 20% | 30% | 40% | 50% | 60% | 70% | 80% | 90% | 100% |
|---|---|---|---|---|---|---|---|---|---|---|---|
| First most important activity | ◯ | ◯ | ◯ | ◯ | ◯ | ◯ | ◯ | ◯ | ◯ | ◯ | ◯ |
| Second most important activity | ◯ | ◯ | ◯ | ◯ | ◯ | ◯ | ◯ | ◯ | ◯ | ◯ | ◯ |
| Third most important activity. | ◯ | ◯ | ◯ | ◯ | ◯ | ◯ | ◯ | ◯ | ◯ | ◯ | ◯ |

## 18. How would you rate your effectiveness in each of these activities identified in Question 15?

| | Very ineffective (1) | Ineffective(2) | Neither effective or ineffective(3) | Effective (4) | Very effective (5) |
|---|---|---|---|---|---|
| First most important activity | ◯ | ◯ | ◯ | ◯ | ◯ |
| Second most important activity | ◯ | ◯ | ◯ | ◯ | ◯ |
| Third most important activity. | ◯ | ◯ | ◯ | ◯ | ◯ |
| Overall effectiveness in your CNC / CMC role | ◯ | ◯ | ◯ | ◯ | ◯ |

## 19. What do you see as the greatest supports / obstacles to your effectiveness?

# Hunter New England Area Health - Survey for Clinical Nurse / Midwife

## 3. Your views about your current role

### 1. In your CNC / CMC role, to what extent can you?

| | Not at all (1) | To a small extent (2) | To some extent (3) | To a considerable extent (4) | To a great extent (5) |
|---|---|---|---|---|---|
| Determine the methods & procedures you use in your work | ○ | ○ | ○ | ○ | ○ |
| Choose what work you will carry out | ○ | ○ | ○ | ○ | ○ |
| Vary how you do your work | ○ | ○ | ○ | ○ | ○ |
| Plan your own work | ○ | ○ | ○ | ○ | ○ |
| Carry out your work in a way you think best | ○ | ○ | ○ | ○ | ○ |

### 2. How true is each of the following statements about your job?

| | Not at all (1) | To a small extent (2) | To some extent (3) | To a considerable extent (4) | To a great extent (5) |
|---|---|---|---|---|---|
| a) I know exactly what is expected of me | ○ | ○ | ○ | ○ | ○ |
| b)I have to keep track of more than one thing at once | ○ | ○ | ○ | ○ | ○ |
| c)I have to solve problems that have no obvious solution | ○ | ○ | ○ | ○ | ○ |
| d)My work requires my undivided attention | ○ | ○ | ○ | ○ | ○ |
| e)I often have too little time to get things done in my job | ○ | ○ | ○ | ○ | ○ |
| f)I have to concerntrate all the time to watch for things going wrong | ○ | ○ | ○ | ○ | ○ |
| g)I have adequate resources to do my job | ○ | ○ | ○ | ○ | ○ |
| h)I know what my responsibilities are | ○ | ○ | ○ | ○ | ○ |
| i)I have to react quickly to prevent problems arising | ○ | ○ | ○ | ○ | ○ |
| j)I have a clear idea of what has to be done in my job | ○ | ○ | ○ | ○ | ○ |
| k)I often have to work extra hours because of staff shortages | ○ | ○ | ○ | ○ | ○ |
| l)I am required to deal with problems that are difficult to solve | ○ | ○ | ○ | ○ | ○ |
| m)People at work make conflicting demands on me | ○ | ○ | ○ | ○ | ○ |
| n)I often have too much work to do in my job | ○ | ○ | ○ | ○ | ○ |
| o)I receive incompatible requests from different people at work | ○ | ○ | ○ | ○ | ○ |
| p)I come across problems in my job I have not encountered before | ○ | ○ | ○ | ○ | ○ |
| q)I have enough administrative support to do my job | ○ | ○ | ○ | ○ | ○ |
| r)I do things at work which are accepted by one person but not by another | ○ | ○ | ○ | ○ | ○ |

## 3. How much of the following do you usually receive from your immediate line manager?

| | None (1) | A little (2) | Some (3) | Quite a lot (4) | A great deal (5) |
|---|---|---|---|---|---|
| Constructive feedback on job performance | ◯ | ◯ | ◯ | ◯ | ◯ |
| Care and concern | ◯ | ◯ | ◯ | ◯ | ◯ |
| Useful information | ◯ | ◯ | ◯ | ◯ | ◯ |
| Help with difficult tasks | ◯ | ◯ | ◯ | ◯ | ◯ |
| Praise and appreciation | ◯ | ◯ | ◯ | ◯ | ◯ |

## 4. How much of the following do you usually receive from your immediate professional manager? (Only answer this question if your Line Manager and Pofessional Manager are different)

| | None (1) | A little (2) | Some (3) | Quite a lot (4) | A great deal (5) |
|---|---|---|---|---|---|
| Constructive feedback on job performance | ◯ | ◯ | ◯ | ◯ | ◯ |
| Care and concern | ◯ | ◯ | ◯ | ◯ | ◯ |
| Useful information | ◯ | ◯ | ◯ | ◯ | ◯ |
| Help with difficult tasks | ◯ | ◯ | ◯ | ◯ | ◯ |
| Praise and appreciation | ◯ | ◯ | ◯ | ◯ | ◯ |

## 5. How much of the following do you usually receive from senior medical staff?

| | None (1) | A little (2) | Some (3) | Quite a lot (4) | A great deal (5) |
|---|---|---|---|---|---|
| Constructive feedback on job performance | ◯ | ◯ | ◯ | ◯ | ◯ |
| Care and concern | ◯ | ◯ | ◯ | ◯ | ◯ |
| Useful information | ◯ | ◯ | ◯ | ◯ | ◯ |
| Help with difficult tasks | ◯ | ◯ | ◯ | ◯ | ◯ |
| Praise and appreciation | ◯ | ◯ | ◯ | ◯ | ◯ |

## 6. How much of the following do you usually receive from your peers and nursing / midwifery colleagues?

| | None (1) | A little (2) | Some (3) | Quite a lot (4) | A great deal (5) |
|---|---|---|---|---|---|
| Constructive feedback on job performance | ◯ | ◯ | ◯ | ◯ | ◯ |
| Care and concern | ◯ | ◯ | ◯ | ◯ | ◯ |
| Useful information | ◯ | ◯ | ◯ | ◯ | ◯ |
| Help with difficult tasks | ◯ | ◯ | ◯ | ◯ | ◯ |
| Praise and appreciation | ◯ | ◯ | ◯ | ◯ | ◯ |

# Hunter New England Area Health - Survey for Clinical Nurse / Midwife

## 7. To what extent do you agree or disagree with each of the following statements?

| | Strongly disagree (1) | Somewhat disagree (2) | Neither agree or disagree (3) | Somewhat agree (4) | Strongly agree (5) |
|---|---|---|---|---|---|
| a)- 1. Senior management (Service managers, SNM, SMM) value my contribution | ○ | ○ | ○ | ○ | ○ |
| - 2. Senior management really care about my wellbeing | ○ | ○ | ○ | ○ | ○ |
| - 3. Senior managementCare about my opinion | ○ | ○ | ○ | ○ | ○ |
| - 4. Senior management actively support me in my role | ○ | ○ | ○ | ○ | ○ |
| b)I am rewarded fairly for the amount of effort I put into my job | ○ | ○ | ○ | ○ | ○ |
| c)This job increases my chances to get ahead in my profession | ○ | ○ | ○ | ○ | ○ |
| d)This job has lived up to expectations I had when I first started | ○ | ○ | ○ | ○ | ○ |
| e)I am rewarded fairly considering the responsibilities that I have | ○ | ○ | ○ | ○ | ○ |
| f)The work I do is very important to me | ○ | ○ | ○ | ○ | ○ |
| g)This job provides the opportunity for me to keep up with new developments related to my profession | ○ | ○ | ○ | ○ | ○ |
| h)Overall I have been disappointed with this job | ○ | ○ | ○ | ○ | ○ |
| i)This job provides me the opportuniy for self-improvement and development | ○ | ○ | ○ | ○ | ○ |
| j)Generally, this job has not been what I thought it would be | ○ | ○ | ○ | ○ | ○ |
| k)I am rewarded fairly in view of my education and background | ○ | ○ | ○ | ○ | ○ |
| l)My experience in this job has been better than I originally expected | ○ | ○ | ○ | ○ | ○ |
| m)I have the opportunity for further advancement in this AHS | ○ | ○ | ○ | ○ | ○ |
| n)This job will open up new opportunities for me in this AHS | ○ | ○ | ○ | ○ | ○ |
| o)I have had the opportunity to participate in collaborations and networks across this AHS | ○ | ○ | ○ | ○ | ○ |
| p)I have had the opportunity to particpate in collaborations and networks statewide and / or nationally | ○ | ○ | ○ | ○ | ○ |
| q)The work I do is very meaningful to me | ○ | ○ | ○ | ○ | ○ |

## 8. In the past five years how many times have you given a presentation about aspects of your work at:

Local forums? [ ]

National forums? [ ]

International forums? [ ]

## 9. In the past five years how many journal publications have you had?

[ ]

## 10. Overall how satisfied are you with your current position?

- ○ Very dissatisfied (1)
- ○ Fairly dissatisfied (2)
- ○ Neither satisfied or dissatisfied (3)
- ○ Fairly satisfied (4)
- ○ Very satisfied (5)

# Hunter New England Area Health - Survey for Clinical Nurse / Midwife

## 4. General attitudes and views

### 1. To what extent do you agree or disagree with each of the following statements?

| | Strongly disagree (1) | Somewhat disagree (2) | Neither agree or disagree (3) | Somewhat agree (4) | Strongly agree (5) |
|---|---|---|---|---|---|
| 1) I have clear written goals and objectives | ○ | ○ | ○ | ○ | ○ |
| 2) I am often bored with my job | ○ | ○ | ○ | ○ | ○ |
| 3) I feel I have all the leadership skills necessary to make a success of my job | ○ | ○ | ○ | ○ | ○ |
| 4) I often feel under excessive stress at work | ○ | ○ | ○ | ○ | ○ |
| 5) I am proud to tell others that I am part of the nursing / midwifery profession | ○ | ○ | ○ | ○ | ○ |
| 6) If a colleague is very busy I often pitch in and help | ○ | ○ | ○ | ○ | ○ |
| 7) My job duties and responsibilities are clearly specified in writing | ○ | ○ | ○ | ○ | ○ |
| 8) I feel I have all the technical and practical knowledge I need to do my job properly | ○ | ○ | ○ | ○ | ○ |
| 9) I often work extra hours as and when necessary | ○ | ○ | ○ | ○ | ○ |
| 10) Most days I am enthusiastic about my job | ○ | ○ | ○ | ○ | ○ |
| 11) Being a nurse / midwife is an important part of who I am | ○ | ○ | ○ | ○ | ○ |
| 12) When I make plans concerning the job, I am certain I can make them work | ○ | ○ | ○ | ○ | ○ |
| 13) I am strongly committed to the values and ideals of my profession | ○ | ○ | ○ | ○ | ○ |
| 14) I am strongly committed to the values and ideals of this AHS | ○ | ○ | ○ | ○ | ○ |
| 15) I am proud to tell others that I work in this AHS | ○ | ○ | ○ | ○ | ○ |
| 16) I often volunteer for things that are not required as part of my job | ○ | ○ | ○ | ○ | ○ |
| 17) I am not happy with my job | ○ | ○ | ○ | ○ | ○ |
| 18) I feel a strong sense of belonging in this AHS | ○ | ○ | ○ | ○ | ○ |
| 19) I often do more than my fair share of the work here | ○ | ○ | ○ | ○ | ○ |
| 20) I worry a lot about my work outside working hours | ○ | ○ | ○ | ○ | ○ |
| 21) I plan to stay in this AHS as long as possible | ○ | ○ | ○ | ○ | ○ |
| 22) My authority and accountability are not clearly specified in my job | ○ | ○ | ○ | ○ | ○ |
| 23) This AHS has a great deal of personal meaning to me | ○ | ○ | ○ | ○ | ○ |
| 24) I am capable of dealing with virtually all problems that come up in my job | ○ | ○ | ○ | ○ | ○ |
| 25) I would be reluctant to leave this AHS | ○ | ○ | ○ | ○ | ○ |
| 26) I find enjoyment in my job | ○ | ○ | ○ | ○ | ○ |
| 27) I often do more than is required of me in my job | ○ | ○ | ○ | ○ | ○ |
| 28) Written rules and guidelines do not exist to direct my work efforts | ○ | ○ | ○ | ○ | ○ |
| 29) I feel myself to be part of this AHS | ○ | ○ | ○ | ○ | ○ |
| 30) I often help my immediate superior by doing things | ○ | ○ | ○ | ○ | ○ |

| | | | | | |
|---|---|---|---|---|---|
| that are not really part of my job | ◯ | ◯ | ◯ | ◯ | ◯ |
| 31) I often have to work very hard on my job | ◯ | ◯ | ◯ | ◯ | ◯ |
| 32) The job provides me the opportunity to expand my professional knowledge | ◯ | ◯ | ◯ | ◯ | ◯ |
| 33) Overall I am satisfied with my job | ◯ | ◯ | ◯ | ◯ | ◯ |
| 34) I plan to leave this AHS as soon as possible | ◯ | ◯ | ◯ | ◯ | ◯ |
| 35) My job is very stressful | ◯ | ◯ | ◯ | ◯ | ◯ |
| 36) I feel I have all the necessary skills to make a success of this job | ◯ | ◯ | ◯ | ◯ | ◯ |
| 37) I feel I have the necessary research skills to do my job | ◯ | ◯ | ◯ | ◯ | ◯ |
| 38) I feel I have the necessary education skills to do my job | ◯ | ◯ | ◯ | ◯ | ◯ |

# Hunter New England Area Health - Survey for Clinical Nurse / Midwife

## 5. Challenges and Innovations in your role

**1. What challenges do you face in your CNC / CMC role? Can you provide an example of how this / these challenges impacts on how you perform your role?**

**2. What innovations have you been able to achieve through your role as CNC/ CMC?**

**3. How have these innovations impacted on:**

Clients?

Service Delivery?

Other health professionals?

**4. What knowledge and skill development do you think you need to acquire to further assist you in fulfilling your CNC / CMC role?**

**5. Do you have any other comments to make in regard to your CNC / CMC role?**
